# Effect of kidney disease on all-cause and cardiovascular mortality in patients undergoing coronary angiography

**DOI:** 10.1080/0886022X.2023.2195950

**Published:** 2023-07-13

**Authors:** Qiang Li, Shanshan Shi, Xiaozhao Lu, Haozhang Huang, Jingru Deng, Weihua Chen, Wenguang Lai, Guoxiao Liang, Yani Wang, Martin Gallagher, Amanda Y. Wang, Jiyan Chen, Jin Liu, Yong Liu

**Affiliations:** a Department of Cardiology, Guangdong Provincial People’s Hospital (Guangdong Academy of Medical Sciences), Southern Medical University, Guangzhou, China; b Guangdong Provincial Key Laboratory of Coronary Heart Disease Prevention, Guangdong Cardiovascular Institute, Guangdong Provincial People’s Hospital (Guangdong Academy of Medical Sciences), Guangzhou, China; c Longyan First Affiliated Hospital of Fujian Medical University, Longyan, China; d The Third Clinical Medical College, Fujian Medical University, Fuzhou, China; e Guangdong Provincial People's Hospital, School of Biology and Biological Engineering, South China University of Technology, Guangzhou, China; f The George Institute for Global Health, UNSW, Sydney, Australia; g Concord Clinical School, University of Sydney, Sydney, Australia

**Keywords:** Kidney disease, all-cause mortality, cardiovascular mortality, coronary angiograph

## Abstract

Acute kidney injury (AKI) occurred in 12.8% of patients undergoing surgery and is associated with increased mortality. Chronic kidney disease (CKD) is a well-known risk for death and cardiovascular disease (CVD). Effects of AKI and CKD on patients undergoing coronary angiography (CAG) remain incompletely defined. The aim of our study was to investigate the relationship between acute and CKD and mortality in patients undergoing CAG. The cohort study included 49,194 patients in the multicenter cohort from January 2007 to December 2018. Cox regression analyses and Fine-Gray proportional subdistribution risk regression analysis are used to examine the association between kidney disease and all-cause and cardiovascular mortality. In the present study, 13,989 (28.4%) patients had kidney disease. During follow-up, 6144 patients died, of which 4508 (73.4%) were due to CVD. AKI without CKD (HR: 1.54, 95% CI: 1.36–1.74), CKD without AKI (HR: 2.02, 95% CI: 1.88–2.17), AKI with CKD (HR: 3.26, 95% CI: 2.90–3.66), and end-stage kidney disease (ESKD; HR: 5.63, 95% CI: 4.40–7.20) were significantly associated with all-cause mortality. Adjusted HR (95% CIs) for cardiovascular mortality was significantly elevated among patients with AKI without CKD (1.78 [1.54–2.06]), CKD without AKI (2.28 [2.09–2.49]), AKI with CKD (3.99 [3.47–4.59]), and ESKD (6.46 [4.93–8.46]). In conclusion, this study shows that acute or CKD is present in up to one-third of patients undergoing CAG and is associated with a substantially increased mortality. These findings highlight the importance of perioperative management of kidney function, especially in patients with CKD.

**Impact Statement**
- **What is already known on this subject?** Acute kidney injury (AKI) occurred in 12.8% of patients undergoing surgery and is linked to a 22.2% increase in mortality. Chronic kidney disease (CKD) is a well-known risk for death and cardiovascular events. Effects of AKI and CKD on patients undergoing coronary angiography (CAG) remain incompletely defined.
- **What do the results of this study add?** This study shows that kidney disease is present in up to one-third of patients undergoing CAG and is associated with a substantially increased mortality. AKI and CKD are independent predicators for mortality in patients undergoing CAG.
- **What are the implications of these findings for clinical practice and/or further research?** These findings highlight the importance of perioperative management of kidney function, especially in patients with CKD.

## Introduction

Chronic kidney disease (CKD) has a high prevalence worldwide, which often leads to poor clinical outcomes and a severe health economic burden [1]. The 2020 USRDS Annual Data Report showed that the prevalence of CKD in patients with cardiovascular disease (CVD) is about 38.3% and is associated with increased mortality [2].

Due to a high burden of CVD, patients with CKD often receive coronary angiography (CAG). Acute kidney injury (AKI) is a common complication after CAG and interventional procedures, with an estimated incidence of up to 12% [3]. It is associated with both kidney and non-kidney adverse outcomes, including the development and progression of CKD, cardiovascular complications, and death [4–6].

Based on the relationship of the aforementioned kidney disease with CVD, revascularization and poor prognosis, AKI and CKD were proven to be risk factors for long-term death [3], but few studies have evaluated how acute and CKDs are associated with all-cause and cardiovascular mortality in patients undergoing CAG. Our main hypothesis was that acute and CKDs would be associated with all-cause and cardiovascular mortality in patients undergoing CAG.

## Methods

### Data collection and study population

We identified cohort from January 2007 to December 2018 among all patients from five tertiary hospital undergoing CAG, and getting admitted subsequently for hospitalization, which from Cardiorenal Improvement II (CIN-II) study (ClinicalTrials.gov NCT05050877). Baseline data were ascertained at the time of admission. AKI was defined during post procedure during the hospital stay. Post discharge, for outcome ascertainment, we used other records for all-cause and cardiovascular mortality. A total of 55,310 patients older than 18 years who underwent CAG, had diagnostic information of AKI and CKD and assessable information of the severity of kidney disease, such as serum creatinine and estimated glomerular filtration rate (eGFR), were initially evaluated. Of these participants, 6116 were excluded because of absent information related to follow-up, being on temporary kidney replacement therapy, or with outliers of eGFR. Thus, 49,194 patients were enrolled in the present study (Supplementary Figure S1). Clinical data of the study patients were obtained from the CIN-II database, mainly including demographic characteristics, medical history, test reports on admission and discharge with medication. Survival information was obtained by cause-specific surveillance data at the regional Center for Disease Control and prevention and public security system, which was completed September 2021 to assess survival through 1 September 2021. The Ethics Committee of the Guangdong Provincial People’s Hospital approved the study (approval no. GDREC2019555H(R1)). It was conducted in accordance with the principles of the Declaration of Helsinki.

### Study outcomes and definitions

All-cause and cardiovascular mortality since the date of CAG were our study outcomes. Cardiovascular mortality was defined as death associated with rheumatic heart disease, hypertension (HT) and its complications, ischemic heart disease, heart failure, inflammatory heart disease, etc. (I100–159, I170–199), diagnosed by the International Statistical Classification of Diseases, Tenth Revision, with any other cause of death treated as a competing risk. Preoperative eGFR and/or prior diagnosis of CKD was used to define CKD. CKD was classified according to the Kidney Disease: Improving Global Outcomes (KDIGO) guidelines: CKD stage 1 or 2 (eGFR 60–90 mL/min/1.73 m^2^), CKD stage 3 (eGFR 30–60 mL/min/1.73 m^2^), CKD stage 4 (eGFR 15–30 mL/min/1.73 m^2^), and CKD stage 5 (eGFR <15 mL/min/1.73 m^2^) [7]. The eGFR was estimated by the Modification of Diet in Renal Disease (MDRD) formula [8,9]. AKI was defined by using the consensus KDIGO criteria as at least a 50% increase within seven days postoperatively and/or a 0.3 mg/dL increase within 48 h postoperatively in serum creatinine level relative to the preoperative reference value. Patients with AKI were stratified according to the maximum change in postoperative serum creatinine level from baseline into three stages: stage 1, a 50% change; stage 2, an 100% increase; and stage 3, a 200% or >4.0 mg/dL increase in serum creatinine level [10].

### Statistical analysis

The study population was divided into five groups: no known kidney disease, AKI without CKD, CKD without AKI, AKI with CKD, and end-stage kidney disease (ESKD). We reported descriptive statistics by means (SD), median (interquartile range (IQR)), or number and percentage when appropriate. We used one-way analysis of variance (ANOVA) to analyze differences between different groups. Categorical variables were compared by Pearson’s Chi-squared test.

To evaluate the association between kidney diseases and all-cause mortality, we used Kaplan–Meier’s estimates to calculate cumulative survival probabilities for all-cause mortality and univariate and multivariate Cox regression analyses, with adjusted hazard ratio (HR) with 95% confidence interval (CI). Characteristic variables with significant baseline differences or clinical significance were used as candidate predictors in the multivariate Cox regression model. In addition, we used the Fine-Gray proportional subdistribution hazards regression analysis to model cardiovascular mortality while treating any other cause of death as a competing risk. First, we analyzed the relationship between the incidence of different kidney diseases during hospitalization and all-cause and cardiovascular mortality. The population-attributable risk percentage (PAR%) related to different kidney diseases was estimated using standard methods [11]. Second, to better examine the prognostic impact of kidney disease, we categorized AKI covariates into stages 1–3, and CKD covariates into CKD stage 1 or 2, CKD stage 3, CKD stage 4, and CKD stage 5 further analysis. *p* Values for trend (*p* trend) were calculated by treating categorical exposure variables as ordinal. We also performed subgroup analyses to investigate whether the risk may be modified by age (≥65 versus <65 years of age), sex, acute myocardial infarction (AMI), and diabetes mellitus (DM). To test whether the pattern of association varies across stratifications, we estimated multiplicative interactions by including the product term (exposure × stratification variable) in the models. All analyses were first unadjusted (model 1), then adjusted for age and sex (model 2), and finally for insurance type, co-morbidity, and contrast media volume (CMV)) (model 3). To assess the robustness of the analytical results under available data only (no imputation), analyses were repeated on all outcomes using multiple regression imputation for the variables included in the regression model correction.

All data analyses were performed using R software (version 4.1.2; R Foundation for Statistical Computing, Vienna, Austria). A two-sided *p* value <.05 indicated significance for all analyses.

## Results

### Baseline characteristics

We analyzed 49,194 patients who underwent CAG between January 2007 and December 2018 (mean age 62.6 ± 11.3 years, 14,591 (29.7%) females), and 13,989 (28.4%) of them had kidney disease. At the time of hospitalization, 235 patients (0.5%) had ESKD and 10,884 patients (22.1%) had CKD. Among the 10,884 patients with CKD, 1419 (13.0%) subsequently developed AKI. After undergoing CAG, 4289 patients (8.7%) developed AKI, of which 2870 (66.9%) had no potential of CKD. Patients with any form of kidney disease were more likely to have co-morbid congestive heart failure (CHF), stroke. Patients with ESKD and CKD are more likely to be older, more likely to have co-morbid HT, DM. Baseline characteristics stratified by the occurrence of kidney disease are presented in Table 1.

**Table 1.** Clinical characteristics for all patients stratified by kidney disease.

Characteristics	Overall (*N* = 49,194)	Patient groups					*p* Value
		No known kidney disease (*N* = 35,205)	AKI without CKD (*N* = 2870)	CKD without AKI (*N* = 9465)	AKI with CKD (*N* = 1419)	ESKD (*N* = 235)	
*Demographic*							
Age, mean (*SD*), years	62.59 (11.29)	60.75 (11.03)	61.27 (9.68)	68.65 (10.28)	70.00 (10.22)	65.07 (10.55)	<.001
Female, no. (%)	14,591 (29.7)	10,072 (28.6)	1178 (41.0)	2789 (29.5)	477 (33.6)	75 (31.9)	<.001
Insurance type, no. (%)							<.001
Urban insurance	36,919 (75.2)	26,499 (75.5)	1960 (68.4)	7182 (76.0)	1087 (76.7)	191 (81.3)	
Rural insurance	5499 (11.2)	3947 (11.2)	495 (17.3)	916 (9.7)	128 (9.0)	13 (5.5)	
Self-paying	6650 (13.6)	4655 (13.3)	410 (14.3)	1352 (14.3)	202 (14.3)	31 (13.2)	
*Medical history*							
AMI, no. (%)	11,332 (23.2)	8166 (23.3)	513 (18.3)	2148 (22.8)	452 (32.1)	53 (22.6)	<.001
HT, no. (%)	25,038 (51.2)	16,741 (47.8)	886 (31.6)	6324 (67.2)	885 (62.9)	202 (86.0)	<.001
DM, no. (%)	15,547 (31.6)	10,123 (28.8)	715 (24.9)	3929 (41.5)	650 (45.8)	130 (55.3)	<.001
AF, no. (%)	4425 (9.1)	2479 (7.1)	588 (21.0)	1124 (11.9)	219 (15.6)	15 (6.4)	<.001
CHF, no. (%)	9467 (19.4)	5312 (15.2)	777 (27.7)	2598 (27.6)	678 (48.2)	102 (43.4)	<.001
Stroke, no. (%)	3169 (6.5)	1933 (5.5)	181 (6.5)	882 (9.4)	157 (11.2)	16 (6.8)	<.001
Cancer, no. (%)	810 (1.7)	549 (1.6)	33 (1.2)	204 (2.2)	21 (1.5)	3 (1.3)	<.001
COPD, no. (%)	1859 (3.8)	1267 (3.6)	67 (2.4)	447 (4.7)	73 (5.2)	5 (2.1)	<.001
PCI, no. (%)	27,771 (56.5)	20,124 (57.2)	858 (29.9)	5807 (61.4)	826 (58.2)	156 (66.4)	<.001
*Laboratory test*							
SCr, mean (SD), %	1.05 (0.55)	0.88 (0.18)	0.85 (0.20)	1.54 (0.50)	1.63 (0.61)	6.04 (2.30)	<.001
eGFR, mean (SD), mL/min/1.73 m^2^	81.25 (26.85)	91.14 (20.42)	92.13 (26.26)	48.19 (12.12)	46.13 (15.91)	10.39 (3.22)	<.001
HGB, mean (SD), g/L	133.41 (17.96)	135.88 (16.34)	132.90 (17.91)	127.04 (19.87)	121.72 (21.00)	97.57 (19.98)	<.001
LDLC, mean (SD), mmol/L	2.92 (1.04)	2.95 (1.04)	2.97 (1.00)	2.79 (1.01)	2.91 (1.05)	2.58 (0.96)	<.001
HDLC, mean (SD), mmol/L	1.07 (0.32)	1.09 (0.32)	1.11 (0.32)	1.02 (0.30)	1.03 (0.31)	0.98 (0.31)	<.001
LVEF, mean (SD), %	57.92 (12.47)	59.12 (11.87)	57.30 (11.03)	55.05 (13.81)	51.13 (13.78)	54.35 (12.68)	<.001
ProBNP, mean (SD), pg/mL	413.10 [93.50, 1610.75]	255.60 [70.00, 1047.00]	860.05 [284.00, 2115.00]	1089.00 [256.20, 3506.00]	3115.00 [1069.50, 7813.48]	9546.00 [2384.50, 25690.00]	<.001
CMV, mean (SD), mL	115.36 (110.61)	115.31 (106.87)	90.35 (76.19)	122.70 (133.24)	117.38 (87.99)	115.22 (73.04)	<.001
*Medication*							
ACEI, no. (%)	18,020 (38.8)	13,521 (40.6)	669 (25.3)	3461 (38.3)	341 (27.6)	28 (13.0)	<.001
ARB, no. (%)	11,241 (24.2)	7787 (23.4)	382 (14.4)	2743 (30.4)	300 (24.3)	29 (13.5)	<.001
Beta-blockers, no. (%)	33,376 (71.9)	24,185 (72.7)	1398 (52.8)	6764 (74.9)	850 (68.9)	179 (83.3)	<.001
Statins, no. (%)	37,797 (81.4)	27,975 (84.0)	1026 (38.7)	7718 (85.5)	905 (73.3)	173 (80.5)	<.001
*Follow-up durations*	5.04 (3.47)	5.37 (3.38)	6.76 (3.57)	5.44 (3.60)	4.94 (3.94)	3.72 (3.35)	

AKI: acute kidney injury; CKD: chronic kidney disease; ESKD: end-stage kidney disease; AMI: acute myocardial infarction; HT: hypertension; DM: diabetes mellitus; AF: atrial fibrillation; CHF: congestive heart failure; COPD: chronic obstructive pulmonary disease; PCI: percutaneous coronary intervention; SCr: serum creatinine; eGFR: estimated glomerular filtration rate; HGB: hemoglobin; LDLC: low-density lipoprotein cholesterol; HDLC: high-density lipoprotein cholesterol; LVEF: left ventricular ejection fraction; ProBNP: pro-B-type natriuretic peptide; CMV: contrast medium volume; ACEI: angiotensin-converting enzyme inhibitor; ARB: angiotensin receptor blocker.

### All-cause and cardiovascular mortality

**Figure 1.**
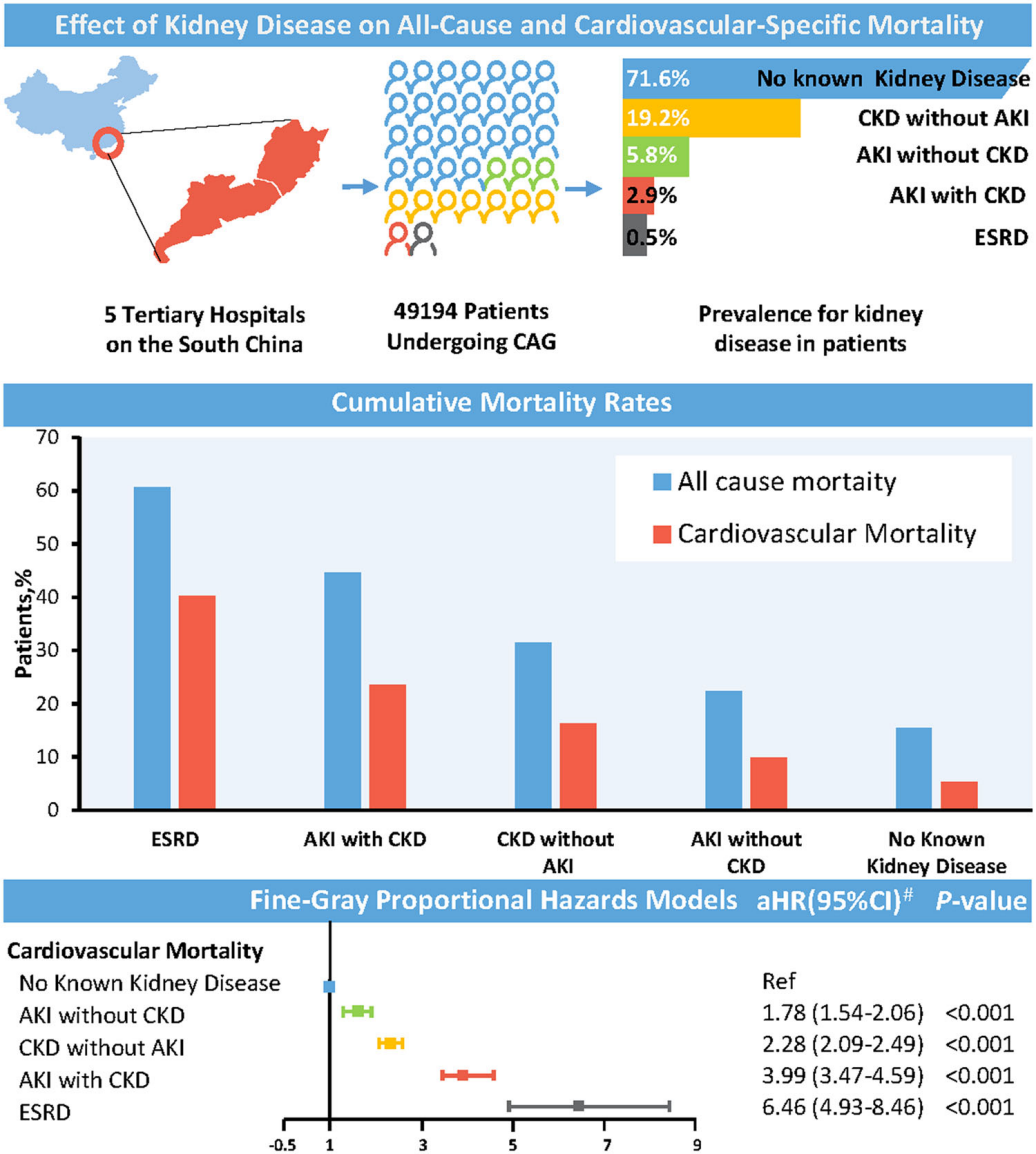
Association between kidney diseases and mortality. CAG: coronary angiography; AKI: acute kidney injury; CKD: chronic kidney disease; ESKD: end-stage kidney disease; HR: hazard ratio; CI: confidence interval. (a) Adjusted for age, sex, insurance type, stroke, hypertension, atrial fibrillation, diabetes mellitus, congestive heart failure, chronic obstructive pulmonary disease, and contrast medium volume.

After a median follow-up of 5.04 years, patients with any form of acute or CKD had significantly higher long-term mortality than patients without kidney disease (Figure 1). For all-cause mortality, patients with no known kidney disease had a mortality rate of 8.6%, whereas patients with any form of kidney disease had a mortality rate of 15.6–36.6%. Patients with AKI but no CKD had the lowest mortality rate (447 of 2870 [15.6%]), while patients with ESKD had the highest mortality rate (86 of 235 [36.6%]). The outcome of cardiovascular mortality was similar to that of all-cause mortality. A higher proportion of patients died of CVD in the AKI without CKD (356 of 2870 [12.4%]), CKD without AKI (1584 of 9465 [16.7%]), AKI with CKD (408 of 1419 [28.8%]), and ESKD groups (70 of 235 [29.8%]) compared with no known kidney disease group (2090 of 35,205 [5.9%]).

**Figure 2.**
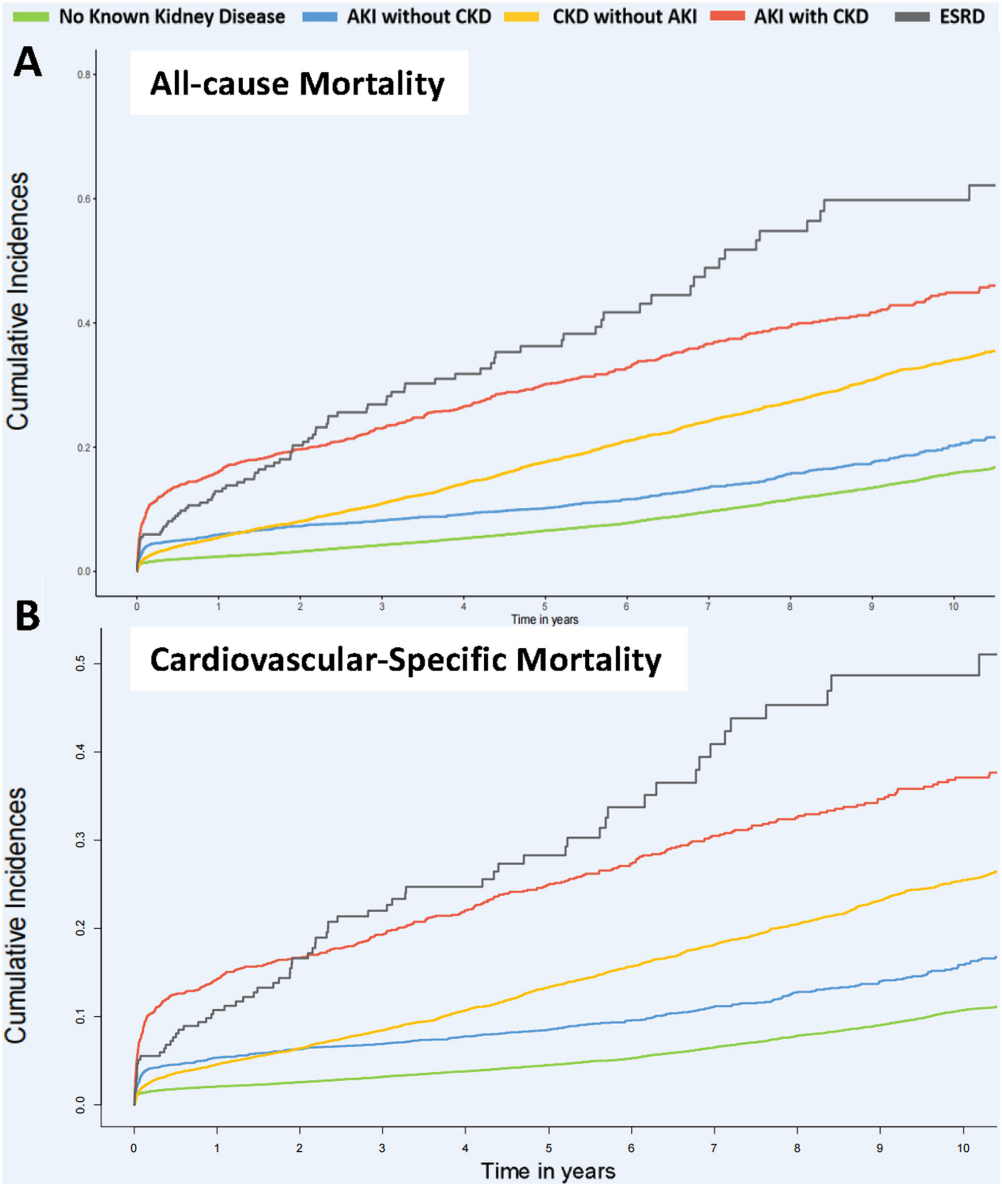
Kaplan–Meier’s survival curves analysis. AKI: acute kidney injury; CKD: chronic kidney disease; ESKD: end-stage kidney disease. (A) All-cause mortality and (B) cardiovascular mortality.

The Kaplan–Meier survival analysis curves for assessing the incidence of all-cause and cardiovascular mortality between groups based on different kidney diseases are shown in Figure 2. There was a statistically significant difference in mortality rate among the five groups (Log-rank *p*< .001).

### Association between kidney diseases and mortality

We studied the prognostic impact of different kidney diseases occurrences on patients who underwent CAG, including cardiovascular mortality and all-cause mortality. Multivariate Cox proportional risk analysis showed that AKI without CKD (HR: 1.54, 95% CI: 1.36–1.74; *p*< .001), CKD without AKI (HR: 2.02, 95% CI: 1.88–2.17; *p*< .001), AKI with CKD (HR: 3.26, 95% CI: 2.90–3.66; *p*< .001), and ESKD (HR: 5.63, 95% CI: 4.40–7.20; *p*< .001) were significantly associated with long-term all-cause mortality. In the multivariable competing risk model, occurrence of different kidney diseases was likewise independently associated with cardiovascular mortality: AKI without CKD (HR: 1.78, 95% CI: 1.54–2.06; *p*< .001), CKD without AKI (HR: 2.28, 95% CI: 2.09–2.49; *p*< .001), AKI with CKD (HR: 3.99, 95% CI: 3.47–4.59; *p*< .001), and ESKD (HR: 6.46, 95% CI: 4.93–8.46; *p*< .001). The multivariate Cox proportional risk model and multivariable competing risk model were adjusted for confusion factors (age, sex, insurance type, HT, AF, DM, stroke, chronic obstructive pulmonary disease, CHF, and CMV; Table 2).

**Table 2.** Association between kidney diseases and mortality.

Variables	Model 1		Model 2		Model 3		PAR, %
	HR (95% CI)	*p* Value	HR (95% CI)	*p* Value	HR (95% CI)	*p* Value	
*All-cause mortality*							
No known kidney disease	Ref.	–	Ref.	–	Ref.	–	Ref.
AKI without CKD	1.44 (1.30–1.59)	<.001	1.53 (1.39–1.69)	<.001	1.54 (1.36–1.74)	<.001	1.54
CKD without AKI	2.54 (2.41–2.69)	<.001	1.84 (1.74–1.95)	<.001	2.02 (1.88–2.17)	<.001	2.02
AKI with CKD	4.30 (3.91–4.73)	<.001	3.07 (2.78–3.38)	<.001	3.26 (2.90–3.66)	<.001	3.26
ESKD	5.93 (4.79–7.35)	<.001	4.93 (3.98–6.12)	<.001	5.63 (4.40–7.20)	<.001	5.63
*Cardiovascular mortality*							
No known kidney disease	Ref.	–	Ref.	–	Ref.	–	
AKI without CKD	1.65 (1.47–1.85)	<.001	1.72 (1.53–1.93)	<.001	1.78 (1.54–2.06)	<.001	1.78
CKD without AKI	2.61 (2.44–2.79)	<.001	2.06 (1.92–2.21)	<.001	2.28 (2.09–2.49)	<.001	2.28
AKI with CKD	4.77 (4.27–5.33)	<.001	3.71 (3.31–4.17)	<.001	3.99 (3.47–4.59)	<.001	3.99
ESKD	6.14 (4.83–7.79)	<.001	5.44 (4.29–6.91)	<.001	6.46 (4.93–8.46)	<.001	6.46

AKI: acute kidney injury; CKD: chronic kidney disease; ESKD: end-stage kidney disease; HR: hazard ratio; CI: confidence interval; PAR: population-attributable risk.

Model 1: unadjusted. Model 2: adjusted for age and sex. Model 3: adjusted for multiple variables (age, sex, insurance type, stroke, hypertension, atrial fibrillation, diabetes mellitus, congestive heart failure, chronic obstructive pulmonary disease, and contrast medium volume).

PAR% estimates the percentage of death in a population that is attributable to the exposure. All-cause mortality PAR% indicate that the exposure accounted for 2.02% among the CKD without AKI group and 3.26% among the AKI with CKD group, but just 1.54% among the AKI without CKD group. Both in all-cause and cardiovascular mortality analyses, AKI with CKD patients had higher PAR% than AKI without CKD and CKD without AKI patients. They consistently peak in patients with ESKD (5.63% and 6.46%).

### Association between kidney disease stratified by severity stages and mortality

Compared with patients with no known kidney disease, risks for all-cause and cardiovascular mortality were proportional to the severity of the kidney disease. Among patients who developed AKI, adjusted HR (95% CI) for all-cause mortality was significantly increased in patients with stage 1 (1.55; 1.35–1.77), stage 2 (1.99; 1.03–3.84), and stage 3 (6.81; 3.05–15.19) kidney injury (*p* trend <.001). Among patients with CKD, the adjusted HR (95% CI) for all-cause mortality was 1.50 (1.26–1.78), 2.15 (1.99–2.33), 3.66 (3.21–4.17), and 5.98 (4.66–7.69) for patients with CKD stage 1 or 2, CKD stage 3, CKD stage 4, and CKD stage 5, respectively (*p* trend <.001). Similarly, compared with patients with no known kidney disease, adjusted HR (95% CI) for cardiovascular mortality was significantly increased in patients with AKI stage 1 (1.80; 1.53–2.11), AKI stage 2 (2.13; 0.96–4.75), AKI stage 3 (10.36; 3.72–28.89) and CKD stage 1 or 2 (1.48; 1.19–1.85), CKD stage 3 (2.45; 2.23–2.70), CKD stage 4 (4.52; 3.87–5.28), and CKD stage 5 (6.81; 5.15–9.00). Additionally, the risk for death tended to increase with growing severity stages of kidney disease (all *p* trend <.001; Table 3). Multiple imputation did not change any of the conclusions (Supplementary Tables S1 and S2).

**Table 3.** Association between kidney disease stratified by severity stages and mortality.

Variables	Model 1		Model 2		Model 3		*p* Trend
	HR (95% CI)	*p* Value	HR (95% CI)	*p* Value	HR (95% CI)	*p* Value	
*All-cause mortality*							
*No known kidney disease*	Ref.	–	Ref.	–	Ref.	–	<.001
AKI							
Stage 1	1.44 (1.29–1.60)	<.001	1.54 (1.38–1.72)	<.001	1.55 (1.35–1.77)	<.001	
Stage 2	2.00 (1.18–3.37)	.010	2.02 (1.19–3.42)	.009	1.99 (1.03–3.84)	.040	
Stage 3	5.10 (2.43–10.71)	<.001	4.85 (2.31–10.19)	<.001	6.81 (3.05–15.19)	<.001	
CKD							
Stage 1 or 2	2.66 (2.32–3.04)	<.001	1.57 (1.37–1.80)	<.001	1.50 (1.26–1.78)	<.001	
Stage 3	2.64 (2.48–2.81)	<.001	1.94 (1.82–2.07)	<.001	2.15 (1.99–2.33)	<.001	
Stage 4	4.52 (4.04–5.04)	<.001	3.46 (3.09–3.87)	<.001	3.66 (3.21–4.17)	<.001	
Stage 5	6.13 (4.93–7.63)	<.001	5.10 (4.10–6.35)	<.001	5.98 (4.66–7.69)	<.001	
*Cardiovascular mortality*							
*No known kidney disease*	Ref.	–	Ref.	–	Ref.	–	<0.001
AKI							
Stage 1	1.62 (1.43–1.83)	<.001	1.70 (1.50–1.92)	<.001	1.80 (1.53–2.11)	<.001	
Stage 2	2.45 (1.37–4.38)	.002	2.48 (1.38–4.48)	.003	2.13 (0.96–4.75)	.063	
Stage 3	7.88 (3.43–18.11)	<.001	7.61 (3.19–18.11)	<.001	10.36 (3.72–28.89)	<.001	
CKD							
Stage 1 or 2	2.32 (1.97–2.74)	<.001	1.58 (1.34–1.88)	<.001	1.48 (1.19–1.85)	<.001	
Stage 3	2.75 (2.55–2.95)	<.001	2.19 (2.03–2.36)	<.001	2.45 (2.23–2.70)	<.001	
Stage 4	4.90 (4.32–5.55)	<.001	4.02 (3.54–4.57)	<.001	4.52 (3.87–5.28)	<.001	
Stage 5	6.27 (4.92–8.00)	<.001	5.59 (4.38–7.14)	<.001	6.81 (5.15–9.00)	<.001	

AKI: acute kidney injury; CKD: chronic kidney disease; HR: hazard ratio; CI: confidence interval.

Model 1: unadjusted. Model 2: adjusted for age and sex. Model 3: adjusted for multiple variables (age, sex, insurance type, stroke, hypertension, atrial fibrillation, diabetes mellitus, congestive heart failure, chronic obstructive pulmonary disease, and contrast medium volume).

## Discussion

In patients receiving CAG, long-term all-cause and cardiovascular mortality was significantly higher in patients with acute or CKD than patients with no known kidney disease. Compared to patients with no known kidney disease, patients with CKD combined with AKI have more than threefold increased all-cause mortality and about fourfold higher cardiovascular mortality. The severer the kidney disease, the higher mortality rate it has, especially death due to CVD.

AKI after CAG is independently associated with cardiovascular mortality, especially in patients with CKD. Even in patients with stage 1 AKI who were not even considered to have true organ damage in clinical practice, the adjusted HR for cardiovascular mortality was increased by 80% compared with patients without kidney disease. In addition, patients with severe CKD prior to admission were more likely to develop AKI during hospitalization than patients with mild and moderate CKD. Cardiovascular mortality was higher in patients with CKD combined with AKI than patients with AKI alone in all periods. In the analysis for all-cause mortality, the results were similar.

CKD is a growing public health problem that affects millions of people worldwide. According to 2013 data from the US Renal Data System, an estimated 43% and 15% of patients with CKD experience heart failure and AMI in their life (compared with healthy individuals: 18.5% and 6.4%, respectively). In addition, CVD is the top cause of death in hemodialysis patients with CKD. The mortality rate from CVD is estimated to be twice as high in patients with stage 3 CKD and three times higher in patients with stage 4 CKD than in healthy subjects [12]. It is certain that the main cause of death in patients with CKD is CVD and patients with underlying kidney disease are at higher risk of long-term adverse prognosis and mortality after CAG [13].

AKI is commonly presented after CAG, and patients with combined kidney disease are more likely to have a higher risk of AKI, significantly increased long-term prognosis and mortality. A recent study reported a strong association between AKI and subsequent cardiovascular events, with an 86% increased risk of cardiovascular-specific death [14]. Another study showed that stage 1 AKI after CAG increased the risk of death twofold compared to no AKI, while stage 2 or 3 AKI increased the risk of death approximately fourfold [6]. Other studies have shown that patients experiencing AKI after major surgery have a higher risk of myocardial infarction, heart failure, stroke, and death from any cause [15–18]. AKI is mainly associated with the use of intraoperative contrast agents, which can cause irreversible damage to kidney function, increase the number of hospital days and costs, and bring about varying degrees of short-term and long-term prognostic damage [19].

In our study, we found that post-contrast patients have high cardiovascular mortality associated with AKI and CKD. This finding has important implications for the preoperative and perioperative management of patients undergoing contrast and percutaneous coronary intervention (PCI). This is similar to the findings of Huber et al. who found that patients after vascular or cardiothoracic surgery had CKD-AKI associated cardiovascular-specific deaths [20]. The main factor contributing to this outcome may be related to the patient’s level of inflammation. Studies suggest that the accumulation of uremic toxins from kidney pathology leads to chronic inflammation and increased oxidative stress, leading to the development of CVD as the damaged endothelium eventually declines [21]. When kidney function declines, retention of late glycosylation end products and pro-oxidants leads to oxidative damage, which may contribute to activation of monocytes and stimulation of inflammatory responses [22,23]. As the CKD stage progresses, plasma inflammation levels continue to rise [22]. The occurrence of AKI, on the other hand, is also capable of bringing about high inflammation levels [24]. The progression of AKI to CKD also has a role for inflammatory factors, which may have an influence on the evolution of the poor prognosis that causes cardiovascular-specific death [25]. Similarly, persistent low-grade inflammation is now considered a prominent, even inherent feature of ESKD, associated with CVD [26]. Persistent low-grade inflammation can act as a catalyst [27], thereby exacerbating atherosclerosis and CVD through enhanced proteolytic metabolism, endothelial dysfunction, and vascular calcification, further turning to cardiovascular mortality.

Therefore, efforts must first focus on AKI prevention during contrast procedures, mitigating further injury when AKI has already occurred, and promoting kidney recovery in patients with established AKI. In addition, we should focus on patients with CKD combined with AKI. The indications to perform contrast procedures in people with CKD and prone to AKI should be fully evaluated and a feasible plan should be established, such as Goal-Directed Intraoperative Management [28]. Although our study did not examine the prognostic differences between patients with acute or chronic kidney insufficiency who underwent CAG versus those who did not, the purpose of this article is to alert clinicians to the importance of assessment of kidney function and intervention in patients undergoing CAG.

We acknowledge that the present study has limitations. First, it is common to all retrospective studies that bias in the results, but our study has attempted to increase the internal validity of the competing risks model through the use of multivariate adjustment and the assessment of model discrimination on validation data sets. Second, our study only focused on the south China, but it was conducted in five tertiary hospitals, representing the entire Chinese sample to some extent. The third point is that we did not examine the GFR directly but estimated it from the serum creatinine. However, it was estimated by the MDRD formula and has been widely used and accepted in previous studies.

## Conclusions

This large multi-center study shows that up to one third of patients undergoing CAG have combined acute and CKD and is associated with a substantially increased mortality. The more serious the kidney disease is, the higher the all-cause and cardiovascular mortality is. This demonstrates the importance of enhanced risk assessment for kidney injury, surveillance of kidney function in the perioperative period and early intervention, especially in patients with CKD.

## Supplementary Material

Supplemental Material

## Data Availability

The data that support the findings of this study are available from the corresponding author upon reasonable request.
