# Supplementary material for: Effect of kidney disease on all-cause and cardiovascular mortality in patients undergoing coronary angiography
Source: Ren Fail. 2023 Jul 13;45(1):2195950. doi: 10.1080/0886022X.2023.2195950 (PMC10348025; doi:10.1080/0886022X.2023.2195950)
Supplement: Supplemental Material [file IRNF_A_2195950_SM1896.pdf]

Table S1. Comparison of Baseline Data Before and After Multiple Regression Interpolation.

**Characteristics**	**No Imputation (N=49 194)**	**Imputation (N=49 194**
Insurance type, No. (%)		
Urban insurance	36919 (75.2)	36919 (75.2)
Rural insurance	5499 (11.2)	5499 (11.2)
Self-paying	6650 (13.6)	6650 (13.6)
HT, No. (%)	25038 (51.2)	25038 (51.2)
AF, No. (%)	4425 (9.1)	4425 (9.1)
CHF, No. (%)	9467 (19.4)	9467 (19.4)
DM, No. (%)	15547 (31.6)	15547 (31.6)
Stroke, No. (%)	3169 (6.5)	3169 (6.5)
COPD, No. (%)	1859 (3.8)	1859 (3.8)
CMV, mean (SD), ml	115.36 (110.61)	115.36 (110.61)

Abbreviation: HT, hypertension; AF, atrial fibrillation; CHF, congestive heart failure; DM, diabetes mellitus; COPD, chronic obstructive pulmonary disease; CMV, contrast medium volume.

Information Classification: General

Table S2. Association Between Kidney Diseases and Mortality After Multiple Regression Imputation.

	Model 1		Model 2		Model 3	
**Variables**	**HR (95%CI)**	***P*-value**	**HR (95%CI)**	***P*-value**	**HR (95%CI)**	***P*-value**
**All-Cause Mortality**						
No known kidney disease	Ref	-	Ref	-	Ref	-
AKI without CKD	1.44 (1.30-1.59)	<0.001	1.53 (1.39-1.69)	<0.001	1.43 (1.29-1.58)	<0.001
CKD without AKI	2.54 (2.41-2.69)	<0.001	1.84 (1.74-1.95)	<0.001	1.72 (1.62-1.83)	<0.001
AKI with CKD	4.30 (3.91-4.73)	<0.001	3.07 (2.78-3.38)	<0.001	2.58 (2.34-2.85)	<0.001
ESRD	5.93 (4.79-7.35)	<0.001	4.93 (3.98-6.12)	<0.001	4.44 (3.58-5.51)	<0.001
**Cardiovascular-Specific Mortality**						
No known kidney disease	Ref	-	Ref	-	Ref	-
AKI without CKD	1.65 (1.47-1.85)	<0.001	1.72 (1.53-1.93)	<0.001	1.59 (1.41-1.79)	<0.001
CKD without AKI	2.61 (2.44-2.79)	<0.001	2.06 (1.92-2.21)	<0.001	1.87 (1.74-2.01)	<0.001
AKI with CKD	4.77 (4.27-5.33)	<0.001	3.71 (3.31-4.17)	<0.001	3.00 (2.67-3.38)	<0.001
ESRD	6.14 (4.83-7.79)	<0.001	5.44 (4.29-6.91)	<0.001	4.54 (3.58-5.76)	<0.001

Abbreviation: AKI, acute kidney injury; CKD, chronic kidney disease; ESKD, end-stage kidney disease; HR, hazard ratio; CI, confidence interval.

Model 1: unadjusted.

Model 2: adjusted for age and gender.

Model 3: adjusted for multiple variables (Age, Gender, Insurance type, Stroke, Hypertension, Atrial fibrillation, Diabetes mellitus, Congestive heart failure, Chronic obstructive pulmonary disease, Contrast medium volume).

Information Classification: General

Figure S1. Flow Chat.

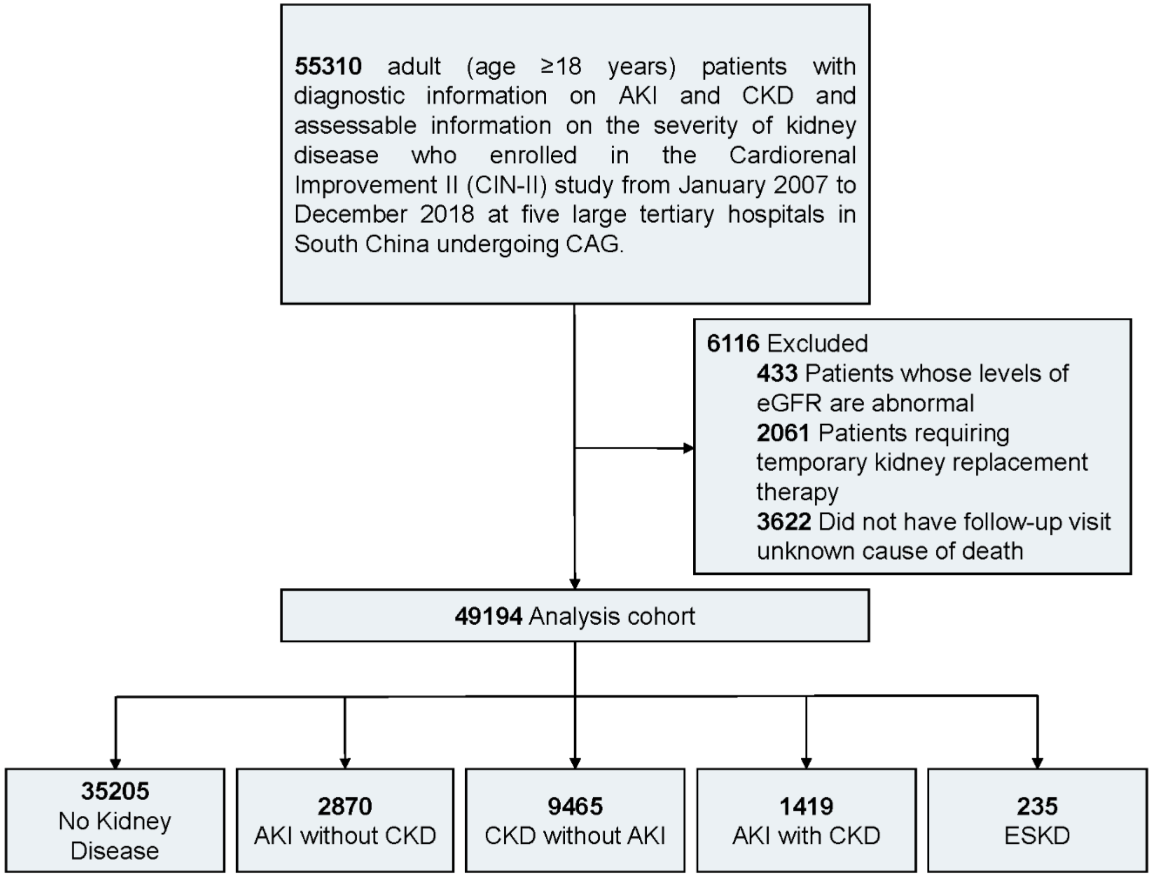

Abbreviation: CAG, coronary angiography; AKI, acute kidney injury; CKD, chronic kidney disease; eGFR, estimated glomerular filtration rate; ESKD, end-stage kidney disease.

Information Classification: General
